# Canine Upper Digestive Tract 3D Model: Assessing Its Utility for Anatomy and Upper Endoscopy Learning

**DOI:** 10.3390/ani14071070

**Published:** 2024-03-31

**Authors:** David Díaz-Regañón, Rosa Mendaza-De Cal, Mercedes García-Sancho, Fernando Rodríguez-Franco, Ángel Sainz, Jesus Rodriguez-Quiros, Concepción Rojo

**Affiliations:** 1 Department of Animal Medicine and Surgery, College of Veterinary Medicine, Complutense University of Madrid, Avda. Puerta de Hierro s/n, 28040 Madrid, Spain; mercgarc@ucm.es (M.G.-S.); ferdiges@ucm.es (F.R.-F.); angelehr@ucm.es (Á.S.); jrquiros@ucm.es (J.R.-Q.); 2 Departmental Section of Anatomy and Embryology, College of Veterinary Medicine, Complutense University of Madrid, Avda. Puerta de Hierro s/n, 28040 Madrid, Spain; rmendaza@ucm.es

**Keywords:** anatomical three-dimensional model, canine endoscopy, three-dimensional printing, veterinary anatomy

## Abstract

**Simple Summary:**

Three-dimensional-printed anatomical models constitute a very useful tool in teaching anatomy and veterinary clinical procedures. In the field of canine gastrointestinal endoscopy, there are no 3D-printed models available other than certain simulators, and none for upper endoscopy. In this study, we aimed to create a 3D-printed anatomical model of the canine upper digestive tract (UDT) that would be valid for both veterinary students in anatomy and internal medicine. The UDT models, printed with molds and silicone casting, were introduced into practical anatomy sessions alongside real specimens. Fifth-year students practiced endoscope handling and anatomical recognition using these 3D models. The results of an anonymous survey indicated positive feedback from both groups of students (138 respondents), confirming the effectiveness of 3D models for learning. The anatomy students most appreciated the ease of manipulation of the 3D model and the better understanding of the size, volume, and topography compared to fresh specimens. Internal medicine students were more enthusiastic, finding the 3D models essential for spatial vision and clinical training. Anatomy students were the most reluctant to completely replace natural specimens, seeing the 3D model as a supplement for learning anatomy, whereas fifth-year students considered it as an essential tool for hands-on clinical endoscopy practice.

**Abstract:**

A teaching strategy using 3D-printed models of the canine upper digestive tract (UDT) for anatomy demonstration and upper endoscopy instruction was evaluated. The canine UDT (esophagus–stomach–duodenum) was scanned and 3D-printed molds were manufactured using silicone casting. First-year students were introduced to these 3D models in practical sessions alongside real specimens. Simultaneously, fifth-year students were trained in endoscope handling and anatomical recognition using 3D specimens. Both groups completed an anonymous survey. Results showed that overall, first-year (*n* = 93) and fifth-year (*n* = 45) students agreed or strongly agreed that the 3D-printed model was effective for learning purposes. In summary, first-year students highlighted an improved understanding of size, volume, topography, and easier manipulation of the 3D model compared to fresh specimens. Fifth-year students were more enthusiastic, finding the 3D model valuable for spatial vision and clinical training. While both groups were against completely replacing the natural UDT with the 3D model, first-year students were more hesitant. These findings suggest that the 3D model of the canine UDT is an effective tool for hands-on training in clinical endoscopy and a valuable, albeit complementary, resource for teaching anatomy and topography.

## 1. Introduction

Three-dimensional-printed models have been shown to be a valuable resource to enhance the teaching of veterinary anatomy [1,2,3,4], including the stomach of dogs [5] and sheep [6]. Three-dimensional printing technology has become a useful resource in veterinary surgery for surgical planning, treatment, and education [7].

Considering the increasing demand for training, limited patients/cadavers, and patient safety, it is necessary to facilitate the learning of medical skills using ethical alternatives [7,8]. Until now, the education program for veterinary medical students at the Veterinary Teaching Hospital Complutense University of Madrid (VTHCUM) has not included simulator or 3D-printed model-based training in flexible endoscopy. Learning has basically been accomplished by observing and assisting in endoscopies.

Medical simulations have been reported to facilitate learning and complement medical education in patient care settings [9], and a few steps have been taken to create reliable customized simulators [10,11,12]. Three-dimensional-printed models have been shown to allow for the development of training exercises in veterinary education, thus enhancing the value of practical laboratories [7,13]. The use of different simulators for the training of veterinary students has been evaluated in various studies on abdominal laparoscopy [14,15,16,17] and gastrointestinal endoscopy [18,19]. To the best of our knowledge, simulation models based on 3D printing to assist veterinary students in learning are still scarce [5,6,13], and none of them relate to training in performing upper endoscopies on dogs.

The aim of the present feasibility study was to assess the perceptions of veterinary anatomy (first-year) and internal medicine (fifth-year) students regarding their learning experience with a flexible silicon model based on 3D-printing technology, in order to validate a comprehensive model for both canine anatomy learning and basic upper endoscopy training. We hypothesize that students will show a high level of acceptance of the 3D model, without advocating its complete replacement of traditional specimens. In addition, we expect to identify differences between first-year and fifth-year students in their perceptions of the characteristics of the model.

## 2. Materials and Methods

### 2.1. Samples and Images

Cadavers were donated by animal shelters in Madrid through collaborative agreements with Complutense University. The upper digestive tract (UDT) (esophagus–stomach–duodenum) was removed from the fresh canine cadavers used for the teaching of anatomy in the veterinary college one day after donation. Ethical permissions were not required.

Immediately after removing, the whole fresh pieces were insufflated with gas. The anatomical position was achieved by manually manipulating the specimen in a tray, giving the duodenum its correct shape. The specimen was laid in anatomic dorsal recumbency in the magnetic resonance imaging (MRI) antenna to obtain digital imaging and communications in medicine (DICOM) images. The MRI scanner was an open low-field MR scanner in “C” configuration (Panorama 0.23TTM R/T, Philips Medical Systems, Eindhoven, The Netherlands), and a SAG T1 FFE3D sequence was chosen.

### 2.2. 3D Model Design and Manufacturing

**Figure 1:**
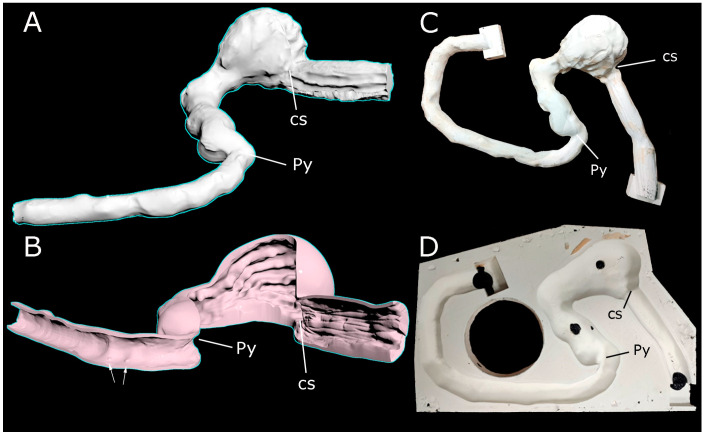
Digital images of the upper digestive tract during processing by 3DMax software. (**A**) Preliminary raw 3D model of the canine stomach obtained from the MRI images. The model is not hollow. (**B**) Sagittal section after merging the external wall and the inner mucosa in a whole 3D model; notice the gastric folds. (**C**) The positive mold of the lumen (with cubes in the ends) was obtained to generate the internal surface mucosa of the silicone model. The cubes permitted a better extraction of the silicone model and a better end resolution after cutting them. (**D**) One of the halves of the negative mold for the external surface wall, with four holes (black spots) to introduce the silicone. Duodenum papillae (arrows); Pylorus (Py); Cardiac sphincter (cs).

Data collected from the DICOM images were processed using D2P software v. 1.03.8. (3D systems Inc., Rock Hill, SC, USA) to a preliminary stereolithography (STL) model of the external wall of the stomach (Figure 1A). A 3D scan to obtain an STL model of the gastric mucosa was made using a Capture™ 3D scanner (3D systems Inc., Rock Hill, SC, USA). Both preliminary meshes were repaired with Autodesk Meshmixer freeware (Autodesk, San Rafael, CA, USA) to obtain meshes without holes. Straightaway, a triangle reduction was carried out for those preliminary models with 3DS Max software (Autodesk, San Rafael, CA, USA). This software was also used for knitting both meshes and obtaining a solid and hollow mesh—the organ with its lumen—of the stomach (Figure 1B). The mucosa surface was placed by repeating the 3D-scanned pattern of the different mucous membranes on their corresponding areas. Afterward, this final model was used to create a positive mold of the lumen of the organ (Figure 1C).

The 3D positive and negative molds of the lumen and external wall (Figure 1C,D) were prepared for 3D printing using 3D Sprint software (3D systems Inc., Rock Hill, SC, USA), a specific slicer software for the printer. The materials for printing were VisiJet Armor M2G-CL and VisiJet M2R-TN resins (3D systems Inc., Rock Hill, SC, USA) for the molds of the external wall and the lumen of the canine stomach, respectively. The parameters used for printing were set by the slicer. Molds were printed using a ProJet MJP 2500 printer (3D systems Inc., Rock Hill, SC, USA) with a build volume of 295 × 211 × 142 mm. Printing time was 52 h and 22 min.

**Figure 2:**
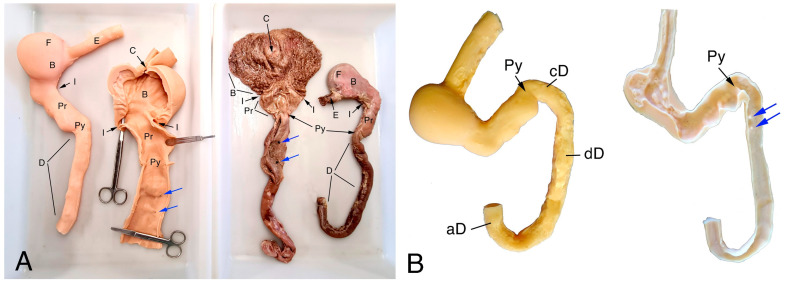
Comparison of 3D-printed models and real organs as displayed to students during the anatomy sessions. (**A**) Whole and opened 3D models (**left**) and real organs (**right**). (**B**) Whole 3D-printed model (**left**) and one of the halves (**right**). The letters and arrows indicate the different anatomical items. Esophagus (E); cardiac sphincter (C); fundus (F); body of the stomach (B); pyloric region (Pr); pylorus (Py); angular incisure (I); duodenum (D): cranial (cD), descendent (dD), and ascendent (aD). Blue arrows point to the duodenum papillae.

The final model of the canine stomach was obtained with platinum-cured silicone (Easyplat 00-30 Flesh, Feroca, Madrid, Spain) using gravity casting. To demold, the obtained model was separated into two halves that were subsequently joined with the same silicone (Figure 2). Prior to obtaining the final model, the wall of the 3D mesh was thickened until a silicone model was obtained that would not break during demolding.

### 2.3. Participants

The respondents were students who participated voluntarily during the teaching activity in the College of Veterinary Medicine. Prior to participation, all students were provided with an informed consent form to ensure that they had access to the information. Subsequently, 95% of the first-year students and 90% of the fifth-year students completed the survey voluntarily. In order to evaluate the impact of knowledge and training on the model evaluation, participants from two groups (first-year, *n* = 93, and fifth-year, *n* = 45, veterinary students) were included. For the first-year veterinary students, the 3D-printed model was included together with the classic canine cadavers during anatomy practice. Fifth-year veterinary students performed an endoscopic exploration on the 3D-printed model before the beginning of programed endoscopic procedures in small animal patients. Fifth-year participants received a half-hour session on basic endoscope handling before training began. All students were asked to complete an anonymous survey based on a Likert rating scale from 1 to 5 (Table 1), where 1 was strongly disagree and 5 was strongly agree. The survey was developed based on those of previous studies [6,20,21].

### 2.4. Statistical Analysis

The statistical analysis of the results was performed using the commercially available statistical software Statgraphics, version 17.2 (XVII Centurion, StatPoint Inc., Herndon, VA, USA). The survey data for each group of students (first-year and fifth-year students) were presented graphically. A first analysis was performed using Mann–Whitney U test, focusing only on the identical questions from both questionnaires (1, 2, 3 on both surveys and questions 12 and 8 on the first-year and fifth-year students’ surveys, respectively). The degree of agreement for each student was calculated as the total sum of their answers. Students’ agreement was then categorized into 5 grades: grade 1 = strongly disagree (total sum ≤ 22), grade 2 = disagree (23 ≤ total sum ≤ 33), grade 3 = neutral (34 ≤ total sum ≤ 44), grade 4 = agree (45 ≤ total sum ≤ 55), and grade 5 = strongly agree (total sum > 55). A percentage of survey respondents was obtained for each agreement category and Likert rating. A second analysis was conducted to compare the degree of agreement (students’ acceptance of the 3D model) between the two courses, also using the Mann–Whitney U test. The significance level was set at *p* < 0.05.

The internal consistency of the questionnaires was assessed using Cronbach’s α (variances of the items). Cronbach’s α = 0.7–0.9 corresponds to an adequate homogeneity of the items. Results for the anatomy and endoscopy questionnaires were Cronbach’s α = 0.8 and Cronbach’s α = 0.7, respectively.

## 3. Results

### 3.1. UDT Manufacturing Results and Preliminary Testing

**Figure 3:**
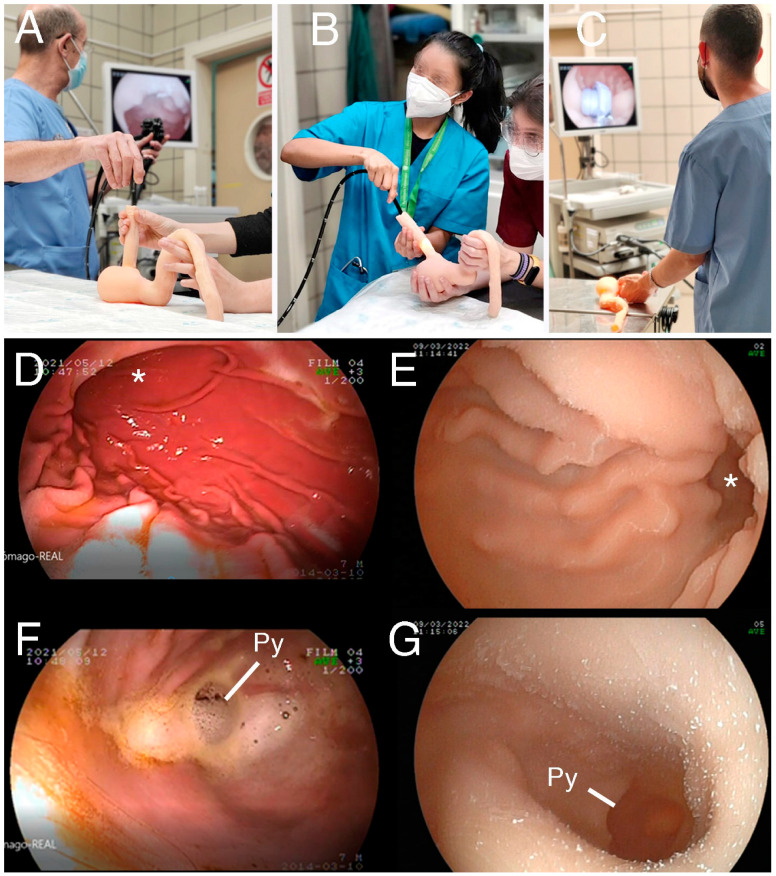
Upper endoscopy training with the 3D-printed models. (**A**) The teacher shows the students the manipulation of the endoscope. (**B**) Students trying the endoscope on the 3D-printed model. Observe how the light of the endoscope helps positioning. (**C**) Student practicing removal of a foreign body from the 3D stomach. (**D**–**G**) Screenshots of the endoscopic images obtained from the stomach, real (**D**,**F**) and 3D-printed (**E**,**G**). (**D**) Gastric folds of the real stomach. Angular incisure (*). (**E**) Gastric folds of the 3D-printed stomach. Angular incisure (*). (**F**) Pyloric region of the real stomach. Pyloric sphincter (Py). (**G**) Pyloric region of the 3D-printed stomach. Pyloric sphincter (Py).

The model was assessed by the professors who specialize in anatomy and endoscopy at the university. The 3D-printed model showed realistic size, texture, and elasticity characteristics, and an acceptable color and appearance. The wall thickness of the final model had to be doubled compared to that of the real organ due to the limitations of the gravity casting technique mainly caused by damages in the model when demolding. Although the silicone model had small imperfections on the external wall such as bulges and rugosities, these did not affect the qualitative characteristics of the gross anatomy (Figure 2). They also did not interfere during the performance of the endoscopy on the 3D-printed model (Figure 3A–C).

The inner surface and cavities of the 3D-printed model reproduced the real ones, showing well-defined gastric folds in the endoscopy images, as well as other anatomical structures such as the angular incisure and the pyloric sphincter (Figure 3D–G). Some minor defects were noted in the 3D-printed model, such as the presence of layer traces (common in 3D printing) on the gastric mucosa and the union line of both valves. The physical characteristics of flexibility and resistance to the passage of the endoscope proved to be realistic. Another interesting advantage of the 3D model was the lack of total opacity of its wall; this allowed the light from the endoscope to be seen from outside, and this fact helped the teachers to show the exact location of the endoscope and the students to localize it easily (see Figure 3B).

### 3.2. Survey Analysis

**Figure 4:**
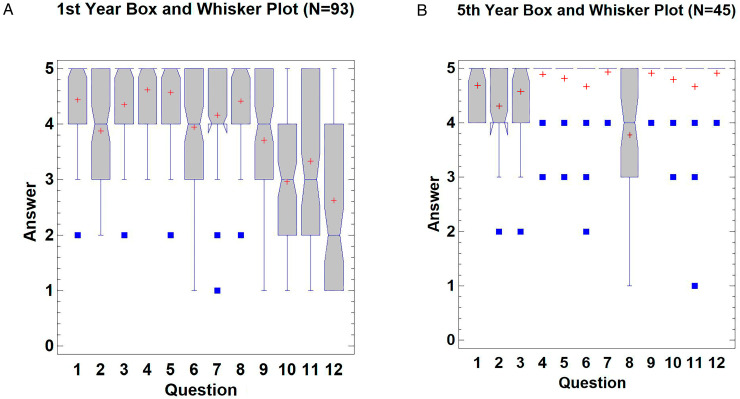
Box and whisker plots of answer values for each question for first-year students’ survey (**A**) and fifth-year students’ survey (**B**). The mean is represented by the red cross, while the median is the blue line that divides the grey bar. To facilitate the identification of the median, there are notches that indicate its location. The blue squares show the outlier results.

A total of 93 first-year students and 45 fifth-year students completed all the questions of their corresponding surveys. The results are shown in Figure 4, SF1_Questionary Answers Anatomy and SF2_Questionary Answers Endoscopy (Supplementary Materials).

First-year students agreed that the external morphology (Likert scale mean ± SD = 4.44 ± 0.73), the internal morphology (3.88 ± 0.88), and the size and the volume (4.35 ± 0.86) of the given 3D-printed model were accurate. They also agreed that the topography and orientation (4.61 ± 0.61), and manipulation of the 3D-printed organ (4.57 ± 0.71) were easier to understand than those of real specimens. For all the physical features of the 3D-printed model, such as rigidity, texture, and color, there was agreement, although regarding the color, some students rated it below 3. The students were asked about the substitution of either the images or the natural UDT for a 3D model on the exam; these results were the most uniformly dispersed of the survey (Figure 4A, questions 10–12).

Fifth-year students showed more agreement than first-year students on their survey with a range of responses between 4 and 5. As shown in Figure 4B, fifth-year students agreed or strongly agreed with most of the questions on the survey, except for question 8, regarding the replacement for studying endoscopy, where more variability in answers was observed.

**Figure 5:**
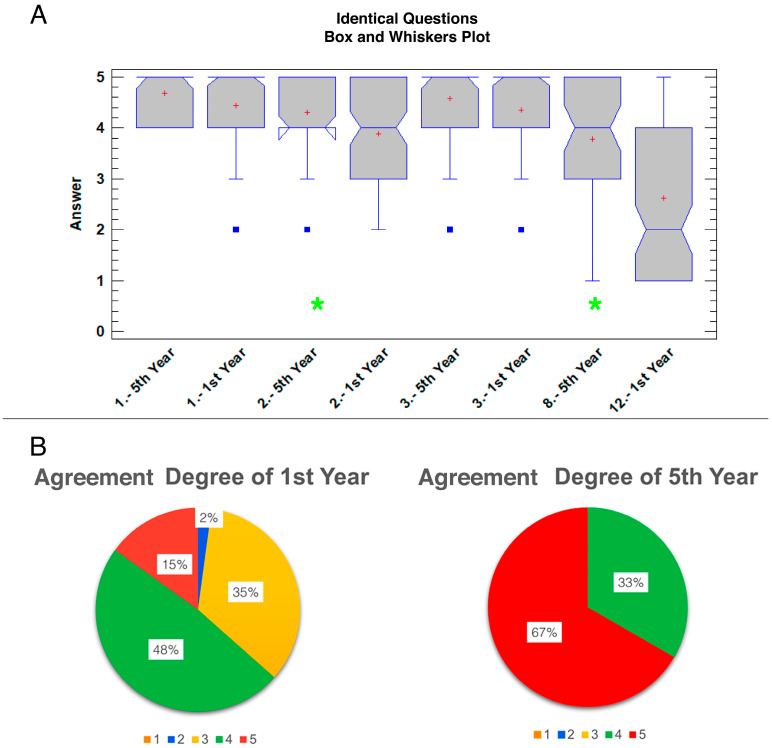
(**A**) Box and whisker plots of comparison of identical questions between both students’ surveys. The mean is represented by the red cross; the median is the blue line that divides the grey bar. To facilitate the identification of the median there are notches that indicate its location. The blue squares show the outliers. Green asterisks represent the significant differences between questions (*p* = 0.005 in question 2; *p* < 0.0001 in question 8 vs. 12). (**B**) Circular charts illustrating the percentage of degree of agreement among first-year and fifth-year students regarding the 3D model; 1—strongly disagree (orange), 2—disagree (blue), 3—neutral (yellow), 4—agree (green), 5—strongly agree (red).

Both surveys shared four questions and the evaluation of the degree of agreement (Figure 5). These questions were 1, 2, and 3 on both surveys and questions 12 and 8 on the first-year and fifth-year students’ surveys, respectively (Table 1). When comparing these identical questions, no statistical differences were found between the courses for questions 1 (*p* = 0.086) and 3 (*p* = 0.124). However, questions 2 (*p* = 0.005) and 8 vs. 12 (*p* < 0.0001) showed statistically significant differences between the answers depending on the year of the student group (Figure 5A). When the agreement degree of the first-year students (median: 4, range: 2–5) was compared with the agreement degree of the fifth-year students (median: 5, range: 4–5), statistically significant differences were also found (*p* < 0.0001). The degree of agreement of the 3D-printed model was ‘agree’ and ‘strongly agree’ for first-year and fifth-year student groups, respectively. The percentage of agreement was 63% for first-year vs 100% for fifth-year students, considering agreement grades 4 and 5 (Figure 5B).

## 4. Discussion

Simulation-based medical education has used 3D-printed models as tools for teaching and training in both human [10,22,23] and veterinary medicine [5,6,8,13,24]. The present work discusses an acceptable training tool for learning anatomy in both basic and clinical contexts. Data from our study are in agreement with those in the review by Azer and Azer [25], showing that the use of a 3D-printed model alongside the real organ enhances students’ skills in spatial visualization of anatomical relationships and allows for easy handling.

### 4.1. High Acceptance of the 3D Model by Students but Not Complete Replacement

Although the anatomy students agreed to work with the 3D-printed model, the total replacement of the real organ was not accepted. This is a common finding when evaluating 3D-printed models, which are suggested to be a good complement but not a substitute for real specimens [26]. Our results are in line with previous experiences in which 3D-printed digestive organs were evaluated by students of veterinary anatomy [6]. Even if the 3D models are prepared in materials similar to tissue, the need for cadavers in teaching seems to be irreplaceable [27].

Results obtained from the survey to fifth-year students showed that the 3D-printed model was highly valued as a tool for training endoscopy. All students strongly agreed that the use of the 3D-printed model helped them with spatial vision and the procedure for taking samples and getting used to it in clinical practice. To the best of our knowledge, there are no previous reports regarding 3D models and their application in small animal flexible gastrointestinal endoscopy. In the present study, the usefulness of 3D models for training veterinary students was similar to that demonstrated in results previously reported for simulators or fresh-frozen canine cadavers [18,19].

Although fifth-year students agreed to work with the 3D-printed model and showed an excellent reception of the model for endoscopy practice, the total replacement of the real specimen was not accepted. This dispersion on answers was normal due to the categorical nature of this question. As described for other simulators, a 3D-printed model is intended as a complementary learning tool [18]. Finally, the use of this asset on a regular basis in endoscopy learning was considered highly useful by the students. This positive perception could be understood because fifth-year students do not have the possibility to practice with the endoscope outside of this 3D-printed model. The same has been reported in the case of human gastroenterologists, who showed enthusiasm about learning endoscopy best through performing procedures as opposed to through classroom sessions, videos, or observing procedures [28].

When the four identical questions posed to all the respondents were compared, the first-year students’ rating was lower, especially on the question regarding the replacement of the natural organ by the model for studying. This could be attributed to the fact that fifth-year students do not practice endoscopy on real patients, so without the 3D-printed model, teaching techniques were perceived as less helpful. Despite manipulating a model that felt similar to the real organ when handled, first-year students expressed a lack of agreement on the total replacement of cadaveric models with 3D models, as previously experienced [6].

### 4.2. Different Learning Context Makes Differences

Students’ recent anatomical memory seems to influence how accurate anatomical details are perceived. Regarding question 1 about external morphology, the first-year students had a real stomach to compare with the 3D-printed model. They also had the most recent anatomical basis. This study, like others before [6,20], showed a good overall appreciation of stomach’s spatial arrangement by the students. Fifth-year students, in contrast, had to remember what the real organ looked like (long-term memory), and indicated that the 3D-printed model looked like the real organ, in broad terms. On question 2, about internal morphology, fifth-year students showed some variability in answers despite remaining at the highest values, as in this case, students could compare the inner surface of the 3D-printed model and that of the real one. Nevertheless, first-year students still seemed to be more judgmental on this question.

This difference in judgment was also reflected in the degree of agreement with survey questions. First-year students showed a greater variability in their degree of agreement. This difference probably corresponded to the short-term/long-term retention of anatomy knowledge and to the different context regarding access to the real organs for both groups. Likewise, the reception was very positive in both student groups. It is probable that applying several materials to produce the models obtaining semi-transparent segments and more realistic haptic experiences will benefit the evaluation of 3D models for learning anatomy [27]. All these differences suggest that first-year students would presumably have more criteria for evaluating whether a 3D-printed model could substitute for the real organ for learning anatomy. This could be explained on the basis that first-year students study the stomach to identify anatomical references, while fifth-year students use the organ for orientation during a clinical procedure, since they are searching for diseases, not for similarities with a normal stomach.

In summary, both groups of students strongly agreed in perceiving the use of the 3D model as an aid in learning canine stomach anatomy. However, perceptions of the learning experience at different stages of the educational process varied in the following ways: (i) first-year students were more critical analyzing similarities to the real organ compared to fifth-year students; (ii) fifth-year students accepted the replacement of the real specimen to a greater extent than first-year students.

### 4.3. Limitations and Future Research

We acknowledge the physical limitations inherent to the printed model itself. The difficulty of sliding an endoscope through a model depends on the material of the 3D stomach and its curing. In this case, the silicone of the inner cavity did not cure properly because it acted as a closed chamber, which did not allow the material to dry. Holding the 3D model during training with the endoscope is necessary, and a suitable receptacle is currently being designed. Further investigations into the proper manufacturing materials required to obtain flexible 3D models for the study of endoscopy should be conducted.

On the other hand, in line with other previous studies of 3D anatomical models [29], our study did not measure learning outcomes. The aim of implementing the 3D model before conducting the endoscopy practice was not to determine whether students would perform a better endoscopy after this learning or not. This would be a future objective in a specific postgraduate course focused on student endoscopic training. Therefore, the model should be evaluated with students in pre-test/post-test experimental studies, comparing a control group´s performance with that of an experimental endoscopy group using only the 3D model.

## 5. Conclusions

The current study developed the first flexible 3D model of the upper digestive tract of the dog as well as its initial application in learning contexts for veterinary students. The anatomical model shows the potential of a 3D-printed model tool for both anatomy learning and medical training. In anatomy learning, the 3D model was a valuable educational tool for students to better understand the topography and to receive adequate haptic feedback and easy handling. In addition, the anatomical 3D model was shown to be an effective tool to teach medical students about the handling of the endoscope and associated procedures, and to perform adequate identifications of the different anatomical parts and structures.

## Figures and Tables

**Table 1:** Student’s survey to assess the educational value of the 3D-printed model.

**A. Veterinary Anatomy (First-Year Students)**
1. The 3D-printed model reproduces the external morphology realistically 2. The 3D-printed model reproduces the internal morphology realistically 3. Size and volume of the organs are easier understood on the 3D-printed model 4. Topography and orientation of the structures are more readily appreciated with the 3D-printed model 5. Hands-on manipulation is easier with the 3D-printed model 6. Compared with the real organ, the color of the 3D-printed model eases the learning of anatomy 7. Compared with the real organ, the texture of the 3D-printed model eases the learning of anatomy 8. Compared with the real organ, the rigidity of the 3D-printed model eases the learning of anatomy 9. I would use again this 3D-printed model for studying anatomy rather than the real organ 10. The 3D-printed model is preferred to real organs for examination 11. The 3D-printed model is preferred to anatomical images for examination 12. The 3D-printed model can replace real specimens in anatomy learning
**B. Upper Digestive Endoscopy (Fifth-year students)**
1. The 3D-printed model reproduces the external morphology realistically 2. The 3D-printed model reproduces the internal morphology realistically 3. Size and volume of the organs are easier understood on the 3D-printed model 4. Topography and orientation of the structures during the endoscopic procedure are more readily appreciated using the 3D-printed model 5. The use of 3D-printed model allows me to better understand the upper gastrointestinal endoscopic examination and the taking of endoscopic biopsies 6. The use of the 3D-printed model allows me to better understand the use of the instruments used for the extraction of foreign bodies 7. The 3D-printed model is useful for endoscopic training of the students 8. The 3D-printed model can replace real specimens in endoscopy learning 9. Do you consider useful the inclusion of 3D-printed models in endoscopy training programs for vet students before the practice? 10. The 3D-printed model is important to complement endoscopy clinical practices 11. The 3D-printed model should always be used in this type of practice 12. I would use this 3D-printed model again to study

Range: 1–5 (1 = strongly disagree; 2 = disagree; 3 = neutral; 4 = agree; 5 = strongly agree).

## Data Availability

All the information obtained in this study is already included in the manuscript and Supplementary Materials. Further inquiries can be directed to the corresponding authors.

## Supplementary Materials

The following supporting information can be downloaded at: https://www.mdpi.com/article/10.3390/ani14071070/s1. Supplementary files: SF1_Questionary Answers Anatomy; SF2_Questionary Answers Endoscopy.
